# Supplementary material for: Pyruvate Dehydrogenase Kinase 1 inhibition mediated oxidative phosphorylation enhancement in cartilage promotes osteoarthritis progression
Source: BMC Musculoskelet Disord. 2023 Jul 20;24:597. doi: 10.1186/s12891-023-06585-6 (PMC10357736; doi:10.1186/s12891-023-06585-6)
Supplement: Supplementary file 1 — Additional file 1. [file 12891_2023_6585_MOESM1_ESM.zip › MF.Gsea.1653627316242/GOMF_P53_BINDING.html]

Details for gene set GOMF\_P53\_BINDING[GSEA]

|  || Dataset | 55457\_collapsed\_to\_symbols.cls.cls #OA\_versus\_normal.cls.cls #OA\_versus\_normal\_repos |
| Phenotype | cls.cls#OA\_versus\_normal\_repos |
| Upregulated in class | normal |
| GeneSet | GOMF\_P53\_BINDING |
| Enrichment Score (ES) | -0.39176103 |
| Normalized Enrichment Score (NES) | -1.515271 |
| Nominal p-value | 0.014754098 |
| FDR q-value | 0.17772105 |
| FWER p-Value | 1.0 |
Table: GSEA Results Summary

  

Fig 1: Enrichment plot: GOMF\_P53\_BINDING      
 Profile of the Running ES Score & Positions of GeneSet Members on the Rank Ordered List

  

| SYMBOL | TITLE | RANK IN GENE LIST | RANK METRIC SCORE | RUNNING ES | CORE ENRICHMENT || 1 | RNF34 | ring finger protein 34 [Source:HGNC Symbol;Acc:HGNC:17297] | 699 | 0.594 | -0.0228 | No |
| 2 | BRD7 | bromodomain containing 7 [Source:HGNC Symbol;Acc:HGNC:14310] | 726 | 0.588 | 0.0065 | No |
| 3 | SMYD2 | SET and MYND domain containing 2 [Source:HGNC Symbol;Acc:HGNC:20982] | 856 | 0.553 | 0.0259 | No |
| 4 | CDKN2AIP | CDKN2A interacting protein [Source:HGNC Symbol;Acc:HGNC:24325] | 1226 | 0.471 | 0.0223 | No |
| 5 | MUL1 | mitochondrial E3 ubiquitin protein ligase 1 [Source:HGNC Symbol;Acc:HGNC:25762] | 1610 | 0.412 | 0.0144 | No |
| 6 | PTTG1IP | PTTG1 interacting protein [Source:HGNC Symbol;Acc:HGNC:13524] | 2012 | 0.360 | 0.0023 | No |
| 7 | TRIAP1 | TP53 regulated inhibitor of apoptosis 1 [Source:HGNC Symbol;Acc:HGNC:26937] | 2163 | 0.342 | 0.0089 | No |
| 8 | RCHY1 | ring finger and CHY zinc finger domain containing 1 [Source:HGNC Symbol;Acc:HGNC:17479] | 2234 | 0.335 | 0.0213 | No |
| 9 | CDK5 | cyclin dependent kinase 5 [Source:HGNC Symbol;Acc:HGNC:1774] | 3400 | 0.211 | -0.0582 | No |
| 10 | TEP1 | telomerase associated protein 1 [Source:HGNC Symbol;Acc:HGNC:11726] | 4210 | 0.136 | -0.1139 | No |
| 11 | BLM | BLM RecQ like helicase [Source:HGNC Symbol;Acc:HGNC:1058] | 4843 | 0.084 | -0.1587 | No |
| 12 | TP53BP1 | tumor protein p53 binding protein 1 [Source:HGNC Symbol;Acc:HGNC:11999] | 4946 | 0.076 | -0.1626 | No |
| 13 | TP73 | tumor protein p73 [Source:HGNC Symbol;Acc:HGNC:12003] | 5222 | 0.055 | -0.1811 | No |
| 14 | EHMT2 | euchromatic histone lysine methyltransferase 2 [Source:HGNC Symbol;Acc:HGNC:14129] | 5507 | 0.032 | -0.2015 | No |
| 15 | TP53 | tumor protein p53 [Source:HGNC Symbol;Acc:HGNC:11998] | 5511 | 0.032 | -0.2000 | No |
| 16 | RNF125 | ring finger protein 125 [Source:HGNC Symbol;Acc:HGNC:21150] | 6049 | -0.008 | -0.2414 | No |
| 17 | MAPKAPK5 | MAPK activated protein kinase 5 [Source:HGNC Symbol;Acc:HGNC:6889] | 6094 | -0.012 | -0.2442 | No |
| 18 | TP63 | tumor protein p63 [Source:HGNC Symbol;Acc:HGNC:15979] | 6274 | -0.025 | -0.2568 | No |
| 19 | TAF9 | TATA-box binding protein associated factor 9 [Source:HGNC Symbol;Acc:HGNC:11542] | 6297 | -0.027 | -0.2571 | No |
| 20 | NOP53 | NOP53 ribosome biogenesis factor [Source:HGNC Symbol;Acc:HGNC:4333] | 6334 | -0.031 | -0.2582 | No |
| 21 | ANKRD1 | ankyrin repeat domain 1 [Source:HGNC Symbol;Acc:HGNC:15819] | 6416 | -0.037 | -0.2626 | No |
| 22 | PSME3 | proteasome activator subunit 3 [Source:HGNC Symbol;Acc:HGNC:9570] | 6903 | -0.072 | -0.2966 | No |
| 23 | MDM2 | MDM2 proto-oncogene [Source:HGNC Symbol;Acc:HGNC:6973] | 7273 | -0.101 | -0.3199 | No |
| 24 | HTT | huntingtin [Source:HGNC Symbol;Acc:HGNC:4851] | 7641 | -0.130 | -0.3416 | No |
| 25 | MUC1 | "mucin 1, cell surface associated [Source:HGNC Symbol;Acc:HGNC:7508]" | 7871 | -0.146 | -0.3516 | No |
| 26 | PRMT5 | protein arginine methyltransferase 5 [Source:HGNC Symbol;Acc:HGNC:10894] | 7904 | -0.148 | -0.3463 | No |
| 27 | HDAC1 | histone deacetylase 1 [Source:HGNC Symbol;Acc:HGNC:4852] | 7911 | -0.148 | -0.3388 | No |
| 28 | POU4F2 | POU class 4 homeobox 2 [Source:HGNC Symbol;Acc:HGNC:9219] | 8292 | -0.178 | -0.3590 | No |
| 29 | TP53BP2 | tumor protein p53 binding protein 2 [Source:HGNC Symbol;Acc:HGNC:12000] | 8336 | -0.181 | -0.3527 | No |
| 30 | DUSP26 | dual specificity phosphatase 26 [Source:HGNC Symbol;Acc:HGNC:28161] | 8424 | -0.187 | -0.3495 | No |
| 31 | TRIM29 | tripartite motif containing 29 [Source:HGNC Symbol;Acc:HGNC:17274] | 8594 | -0.201 | -0.3520 | No |
| 32 | KDM1A | lysine demethylase 1A [Source:HGNC Symbol;Acc:HGNC:29079] | 8608 | -0.202 | -0.3422 | No |
| 33 | DAXX | death domain associated protein [Source:HGNC Symbol;Acc:HGNC:2681] | 8926 | -0.226 | -0.3549 | No |
| 34 | SIRT1 | sirtuin 1 [Source:HGNC Symbol;Acc:HGNC:14929] | 8947 | -0.227 | -0.3443 | No |
| 35 | NUAK1 | NUAK family kinase 1 [Source:HGNC Symbol;Acc:HGNC:14311] | 9190 | -0.247 | -0.3500 | No |
| 36 | PLK3 | polo like kinase 3 [Source:HGNC Symbol;Acc:HGNC:2154] | 9698 | -0.291 | -0.3740 | No |
| 37 | TRIM24 | tripartite motif containing 24 [Source:HGNC Symbol;Acc:HGNC:11812] | 9927 | -0.314 | -0.3750 | Yes |
| 38 | USP10 | ubiquitin specific peptidase 10 [Source:HGNC Symbol;Acc:HGNC:12608] | 9955 | -0.317 | -0.3603 | Yes |
| 39 | SMARCA4 | "SWI/SNF related, matrix associated, actin dependent regulator of chromatin, subfamily a, member 4 [Source:HGNC Symbol;Acc:HGNC:11100]" | 10009 | -0.322 | -0.3472 | Yes |
| 40 | SMARCB1 | "SWI/SNF related, matrix associated, actin dependent regulator of chromatin, subfamily b, member 1 [Source:HGNC Symbol;Acc:HGNC:11103]" | 10117 | -0.333 | -0.3378 | Yes |
| 41 | AXIN1 | axin 1 [Source:HGNC Symbol;Acc:HGNC:903] | 10335 | -0.354 | -0.3358 | Yes |
| 42 | CREBBP | CREB binding protein [Source:HGNC Symbol;Acc:HGNC:2348] | 10881 | -0.414 | -0.3562 | Yes |
| 43 | GATA1 | GATA binding protein 1 [Source:HGNC Symbol;Acc:HGNC:4170] | 10958 | -0.425 | -0.3395 | Yes |
| 44 | MDM4 | MDM4 regulator of p53 [Source:HGNC Symbol;Acc:HGNC:6974] | 11047 | -0.437 | -0.3230 | Yes |
| 45 | GSK3B | glycogen synthase kinase 3 beta [Source:HGNC Symbol;Acc:HGNC:4617] | 11157 | -0.451 | -0.3075 | Yes |
| 46 | HSPD1 | heat shock protein family D (Hsp60) member 1 [Source:HGNC Symbol;Acc:HGNC:5261] | 11427 | -0.492 | -0.3022 | Yes |
| 47 | STK11 | serine/threonine kinase 11 [Source:HGNC Symbol;Acc:HGNC:11389] | 11451 | -0.496 | -0.2776 | Yes |
| 48 | PPP1R13B | protein phosphatase 1 regulatory subunit 13B [Source:HGNC Symbol;Acc:HGNC:14950] | 11589 | -0.513 | -0.2610 | Yes |
| 49 | EHMT1 | euchromatic histone lysine methyltransferase 1 [Source:HGNC Symbol;Acc:HGNC:24650] | 11686 | -0.530 | -0.2402 | Yes |
| 50 | EP300 | E1A binding protein p300 [Source:HGNC Symbol;Acc:HGNC:3373] | 11741 | -0.540 | -0.2157 | Yes |
| 51 | CHD8 | chromodomain helicase DNA binding protein 8 [Source:HGNC Symbol;Acc:HGNC:20153] | 11825 | -0.554 | -0.1926 | Yes |
| 52 | HIF1A | hypoxia inducible factor 1 subunit alpha [Source:HGNC Symbol;Acc:HGNC:4910] | 11833 | -0.556 | -0.1636 | Yes |
| 53 | MSX1 | msh homeobox 1 [Source:HGNC Symbol;Acc:HGNC:7391] | 12054 | -0.609 | -0.1483 | Yes |
| 54 | NTRK3 | neurotrophic receptor tyrosine kinase 3 [Source:HGNC Symbol;Acc:HGNC:8033] | 12157 | -0.632 | -0.1226 | Yes |
| 55 | USP7 | ubiquitin specific peptidase 7 [Source:HGNC Symbol;Acc:HGNC:12630] | 12289 | -0.673 | -0.0969 | Yes |
| 56 | RFWD3 | ring finger and WD repeat domain 3 [Source:HGNC Symbol;Acc:HGNC:25539] | 12427 | -0.717 | -0.0694 | Yes |
| 57 | BRD4 | bromodomain containing 4 [Source:HGNC Symbol;Acc:HGNC:13575] | 12606 | -0.800 | -0.0407 | Yes |
| 58 | TAF1 | TATA-box binding protein associated factor 1 [Source:HGNC Symbol;Acc:HGNC:11535] | 12881 | -1.192 | 0.0015 | Yes |
Table: GSEA details [plain text format]

  

Fig 2: GOMF\_P53\_BINDING      
 Blue-Pink O' Gram in the Space of the Analyzed GeneSet

  

Fig 3: GOMF\_P53\_BINDING: Random ES distribution      
 Gene set null distribution of ES for **GOMF\_P53\_BINDING**
